# 17ß-Estradiol Antagonizes the Down-Regulation of ERα/NOS-3 Signaling in Vascular Endothelial Dysfunction of Female Diabetic Rats

**DOI:** 10.1371/journal.pone.0050402

**Published:** 2012-11-29

**Authors:** Yi Han, Xiaozhen Li, Suming Zhou, Guoliang Meng, Yujiao Xiao, Wen Zhang, Zhuoying Wang, Liping Xie, Zhen Liu, Hui Lu, Yong Ji

**Affiliations:** 1 Department of Geriatrics, the First Affiliated Hospital of Nanjing Medical University, Nanjing, People’s Republic of China; 2 State Key Laboratory of Reproductive Medicine, Laboratory of Cardiovascular Disease and Molecular Intervention, Nanjing Medical University, Nanjing, People’s Republic of China; The Chinese University of Hong Kong, Hong Kong

## Abstract

Previous studies indicated that estrogen could improve endothelial function. However, whether estrogen protects vascular complications of diabetes has yet to be clarified. The study was designed to investigate the action of 17ß-estradiol on vascular endothelium in streptozotocin (STZ)-induced diabetic rats. Ovariectomized female Sprague-Dawley rats were administered with streptozotocin to produce an ovariectomized-diabetic (OVS) model which manifested as dysfunction of aortic dilation and contraction ability. Meanwhile, OVS animals with 17ß-estradiol supplementation significantly improved aortic function. Accordingly, nitric oxide synthase-3 (NOS-3), Akt, PI3K and estrogen receptor α (ERα) protein expression in aorta declined in the OVS group. Such effects were partially restored by estrogen replacement. The presence of 17ß-estradiol similarly counteracted the reduction of cyclic guanosine monophosphate (cGMP), the enhanced expression of inducible NOS (NOS-2) and NO metabolites (nitrite and nitrate), as well as the increase of matrix metalloproteinase-9/tissue inhibitor of metalloproteinase-1 (MMP-9/TIMP-1), which is an index of arterial compliance. 17ß-estradiol could also decrease ROS production in vascular endothelium. In EA hy 926 cells we found that ER antagonist, wortmannin and Akt inhibitor could block improvement effects of 17ß-estradiol. These results strongly suggest that functional impairment of the ERα/NOS-3 signaling network in OVS animals was partially restored by 17ß-estradiol administration, which provides experimental support for estrogen recruitment to improve vascular outcomes in female diabetes after endogenous hormone depletion.

## Introduction

The most important complications of diabetes relate to vascular disease, both in the micro- and macro-vasculature and endothelial dysfunction is implicated in the pathogenesis of diabetic vascular disorders [1]. Reduced endothelial nitric oxide (NO) generation has been well documented in vivo in patients with type 1 or 2 diabetes, leading to the pathogenesis of diabetic vascular damage. A progressive decrease of endothelial nitric oxide synthase (NOS-3) expression, due to long term exposure of high glucose, advanced glycation end-products (AGEs) accumulation, or a combination of both processes, was discovered to be very important in the context of diabetes [2]. We have previously demonstrated that AGEs suppressed NOS-3 activity in endothelial cells both in the short term (within 30 min of incubation), involving a decrease in serine phosphorylation of NOS-3 [2], and in the long term (with hours or days of administration), involving a suppression of NOS-3 protein expression [3].

Multiple biological effects of estrogen have been shown in numerous animal, cellular and molecular models, which support the favorable effects of estrogen on vascular structure, function, and cell signaling. This includes the estrogen-stimulated, short and long term activation of NOS-3, resulting in synthesis of NO [4]. Both endothelial- or platelet-derived NO is an established key regulator of vascular tone and inhibitor of platelet aggregation, and thus a likely target of estrogen for vascular protective modulation, such as the athero-protection and angiogenesis promotion. Furthermore, it seems that estrogen exerts those protective effects via distinct forms of estrogen receptor (ER) α instead of ERβ [4], [5].

NOS-3 protein possesses multiple putative phosphorylation sites, which can be phosphorylated by various protein kinases including Akt and ERK2/1 [6], [7]. On estrogen stimulation, PI3K rapidly phosphorylates the downstream serine/threonine kinase Akt, activated Akt in turn phosphorylates serine 1177 of NOS-3 in endothelial cells [8], [9]. In endothelial cells, Akt activation has also been reported to promote NOS-3 protein expression, which leads to increase of NO production. And cGMP, as a second messenger, represents the bioactive index of NO at the downstream.

**Table 1:** Physiological parameters.

Physiological parameters	SHAM	OVX	OVE	OVS	OVSE
Body weight, g	241.3±9.8	256.7±18.0	227.9±16.6	187.2±35.7*	206.8±17.5*
Uterus weight, g	1.01±0.13	0.56±0.28*	1.01±0.16#	0.38±0.14*	0.89±0.24&
circulating E2, pg/ml	522.3±156.6	304.7±75.3*	629.3±130.7#	242.8±27.2*	699.5±196.8&
Insulin, µg/l	11.62±6.67	8.79±3.96	10.75±1.40	5.58±2.57*	6.36±2.21*
Blood glucose, mmol/L	11.1±3.0	11.6±3.0	11.9±2.8	29.6±5.4*	30.7±3.5*
HbA1c, %	2.1±1.4	2.4±1.6	2.9±2.4	6.2±2.9*	6.0±2.0*
TG, mmol/L	1.21±0.92	1.36±0.99	2.32±0.80	2.39±1.91	2.76±1.12
TC, mmol/L	3.47±2.56	2.01±0.77	4.88±2.27	2.84±1.08	3.23±1.06
LDL, mmol/L	1.46±0.49	1.67±0.63	1.92±0.89	2.34±0.72	1.34±0.13
HDL (mmol/L)	0.54±0.35	0.71±0.45	0.68±0.61	0.44±0.20	0.77±0.16

TG, triglyceride; TC, total cholesterol; LDL, low-density lipoprotein; HDL, high-density lipoprotein; SHAM, sham operated; OVX, bilateral ovariectomized; OVE, ovariectomized+E2 supplemented; OVS, ovariectomized+STZ injected; OVSE, ovariectomized+STZ injected+E2 supplemented.

* *P*<0.05 vs. SHAM;

# *P*<0.05 vs. OVX;

& *P*<0.05 vs.OVS.

However, the signaling mechanisms involved in protective effects of estrogen against diabetic vascular disorders remain unclear. In the present work, we sought to investigate the effect of estrogen on NOS-3 associated vascular function in a streptozotocin (STZ)-induced diabetes model and the underlying mechanisms related to the ERα/NOS-3 signaling network. Additionally, we demonstrated alterations of cGMP, NO metabolites and NOS-2, as well as the arterial stiffness indices MMP-9 and TIMP-1, since the NO pathway in the endothelium and the activation of MMP system play key roles in arterial remodeling [10].

**Figure 1:**
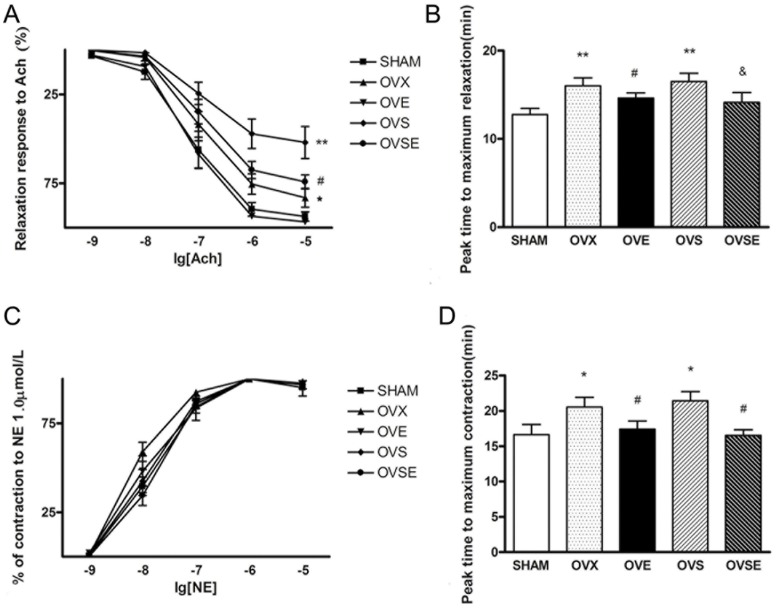
Effects of 17ß-estradiol on thoracic aorta reactivity in diabetic rats with ovariectomy. (A) Concentration-response curves to Ach (10^−9^–10^−5^ mol/L). Aortic rings were pre-contracted with 0.1 µmol/L NE to a maximal level before cumulative Ach addition. Data are expressed as percentage of contraction to 0.1 µmol/L NE; (B) Peak time to maximum relaxation; (C) Concentration-response curves to NE (10^−9^–10^−5^ mol/L); (D) Peak time to maximum contraction in aortic rings isolated from rats in all groups. Data are expressed as mean ± SEM, and from 6 independent experiments in each group. ^*^ *P*<0.05, ^**^ *P*<0.01, vs. SHAM; ^#^ *P*<0.05, vs. OVX; ^&^ *P*<0.05, vs. OVS.

## Materials and Methods

### Animals and Cells

Ethics approval was obtained from the Animal Care and Use Committee of Nanjing Medical University. All experiments were conducted in accordance with the Guide for the Care and Use of Laboratory Animals adopted by the Institutional Animal Care and Use Committee (IACUC). Every effort was made to minimize animal suffering and the number of animals used.

**Figure 2:**
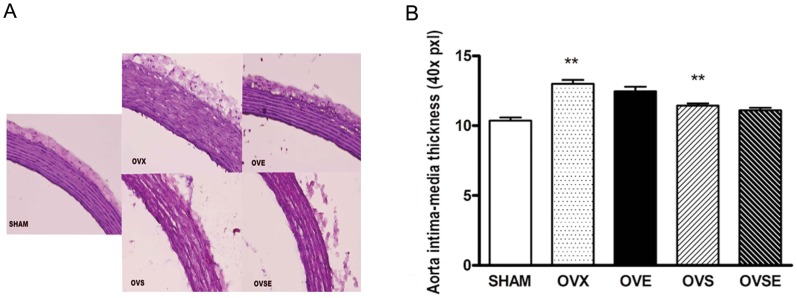
Effects of 17ß-estradiol on aortic histological structure in diabetic rats with ovariectomy. (A)Histological analysis of aortic tissues from all groups of animals withed with hematoxylin -eosin staining, and no differences were observed among groups with regard to vascular integrity. (B)The intima-media thickness was increased in OVX group, and 17β-estradiol reintroduction wiped off this increase. Data are shown as mean ± SEM from 6 sets of independent experiments.^ *^ *P*<0.05 vs. SHAM.

Female SD rats initially weighing 220 to 250 g, were obtained from the Laboratory Animal Center, Nanjing Medical University. Animals were kept in temperature-controlled facilities on a 12-hour light/dark cycle and fed normal chow [11]. Bilateral ovariectomy or sham operation was performed under chloral hydrate anesthesia. Supplementation of 17β-estradiol benzoate (E2, 12 g/kg) or corresponding vehicle with subcutaneous injection was implemented 6 days after ovarian ablation, to allow a recovery from the surgical trauma and a clear-out of the endogenous estrogen [12]. The 17β-estradiol replacement continued for 4 weeks. Meanwhile, STZ (60 mg/kg, i.p., Sigma) was administered to develop a diabetes model. It is important to note that STZ-induced diabetes mellitus has been confirmed to produce experimental vascular endothelial dysfunction [13]. Female SD rats were randomly divided into 5 groups, with 6 in each group, and the animals were sham operated (SHAM), bilateral ovariectomized (OVX), ovariectomized+E2 supplemented (OVE), ovariectomized+STZ injected (OVS), or ovariectomized+STZ injected+E2 supplemented (OVSE) respectively. Animals were euthanized after 4 weeks of 17β-estradiol supplementation. Blood samples were collected to determine level of blood glucose, HbA1c, cholesterol profile, E2 and insulin through biochemical means or radioimmunoassy. Aortas were harvested for evaluation of vaso-contraction and dilation responses, for histology analysis of frozen sections and for western blot analysis of extracted protein. Ovaries (in intact females), uterus were dissected and trimmed of connective tissue and fat to obtain uterus weight.

**Figure 3:**
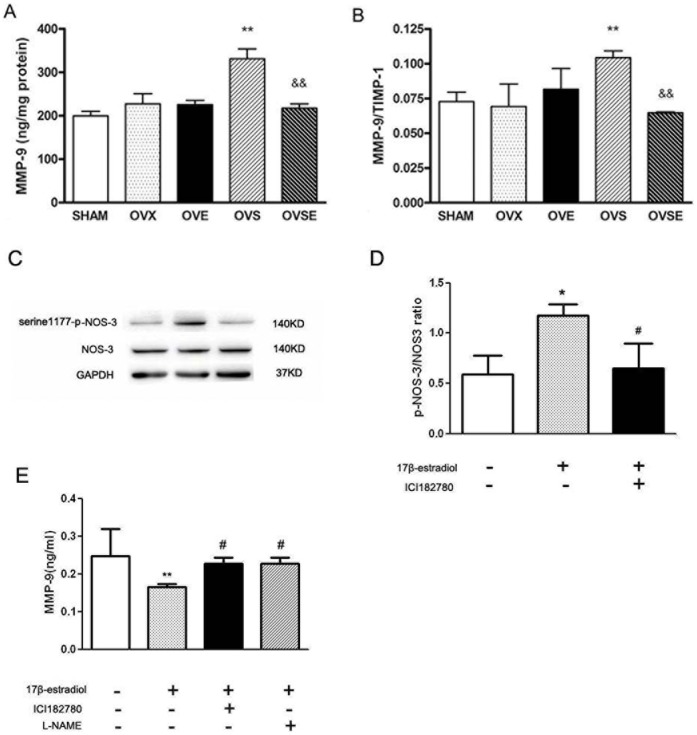
Effects of 17ß-estradiol on MMP-9/TIMP-1. Effect of 17ß-estradiol on vascular MMP-9 contents (A) and vascular MMP-9/TIMP-1 ratio(B) in diabetic rats with ovariectomy. Each sample was measured with ELISA. Data were shown as mean ± SEM from 6 sets of independent experiments. ^**^ *P*<0.01, vs. SHAM; ^&&^ *P*<0.01 vs. OVS. (C, D) Expression of serine1177-p-NOS-3 and NOS-3, (E) Contents of MMP9 in cellular medium in EA hy 926 cells pre-incubated with specific ER antagonist (ICI 182780, 1 µM) or NOS-3 inhibitor (L-NAME, 100 µM) before 17ß-estradiol (100 nM) stimulation. Western blot analysis was performed using each relevant antibody. GAPDH was performed as a loading control. ^*^ *P*<0.05, vs. control; ^#^ *P*<0.05, vs 17ß-estradiol stimulation alone.

EA hy 926 cells were obtained from American type culture collection (ATCC). Cells were cultured in Dulbecco’s modified Eagle’s medium (DMEM) with 10% v/v fetal bovine serum (FBS), penicillin (100 U/mL) and streptomycin (100 mg/mL) in a 95% O_2_: 5% CO_2_ humidified atmosphere at 37°C. Confluent cells (85%–90%) were pre-incubated with or without ICI 182780 (specific ER antagonist, 1 µM, Toc-ris), wortmannin(specific PI3K inhibitor, 200 nM, Beyotime), AKT inhibitor (20 µM, Calbiochem) or L-NAME (NOS-3 inhibitor, 100 µM, Sigma) for 30 minutes before 17ß-estradiol (100 nM) stimulation for 24 hours.

**Figure 4:**
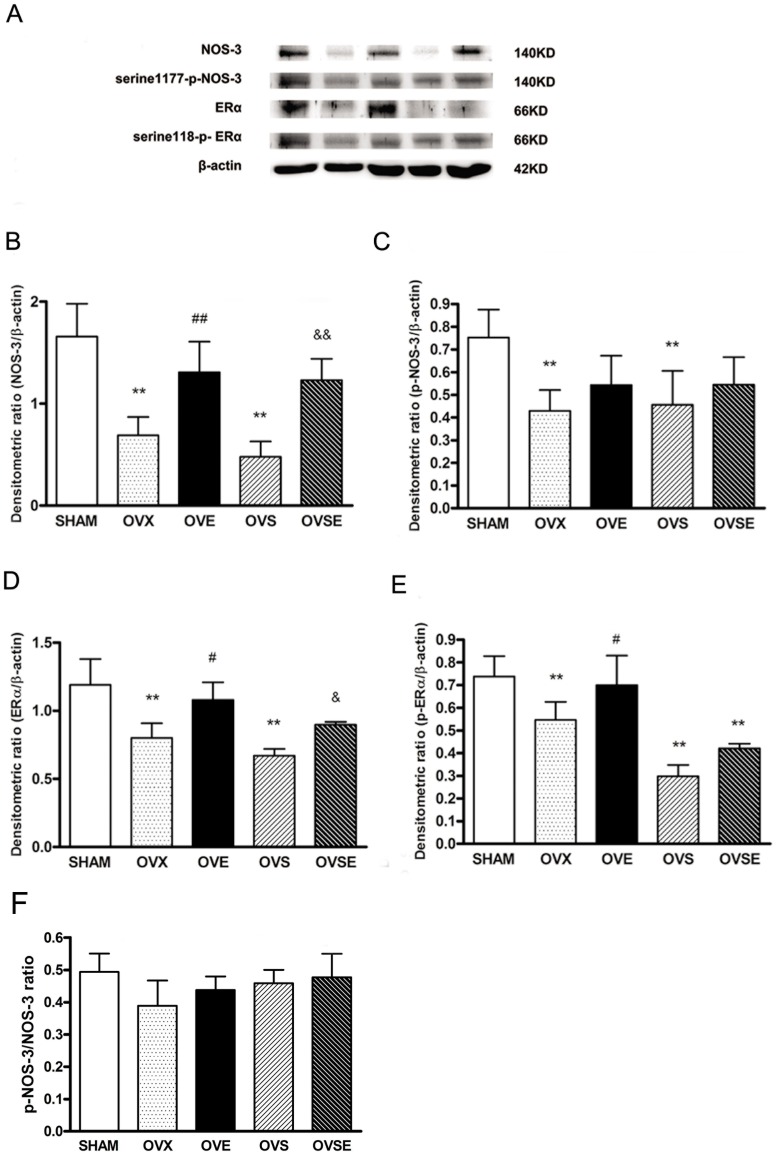
Effects of 17ß-estradiol on vascular protein expression of NOS-3 and ERα in diabetic rats with ovariectomy. (A, B) Expression of NOS-3 (A, C)serine1177-p-NOS-3, (A, D)ERα, (A,E) serine118-p- ERα (F) ratio of p-NOS-3/NOS-3 in vascular isolated from all groups of animals.Western blot analysis was performed using each relevant antibodies. β-Actin was performed as a loading control. Data were shown as mean ± SEM from 6 sets of independent experiments. ^**^ *P*<0.01, vs. SHAM; ^#^ *P*<0.05,^ ##^ *P*<0.01, vs. OVX; ^&^ *P*<0.05, ^&&^ *P*<0.01, vs. OVS.

### Isolated Organ Bath Experiments

Experiments were performed on isolated aortic rings excised from female SD rats. The aorta was carefully removed, cleaned of fat and connective tissue, and cut into 3 to 4 mm rings. Vessels were suspended in 4 ml organ baths containing Kreb’s solution at 37°C, continuously bubbled with 95% O_2_ and 5% CO_2_. The Kreb’s solution had the following composition (mmol/L): NaCl 118, KCl 4.7, KH_2_PO_4_ 1.2, MgSO_4_ 1.1, CaCl_2_ 2.5, NaHCO_3_ 25, and glucose 5.5 (pH7.4). The rings were connected to isometric tension transducers coupled with a digital recording system (DMT). Experiments were carried out on tissues pre-contracted with noradrenaline (NE 0.1 µmol/L) to elicit maximal contractions, then cumulative concentration-response curves for acetylcholine (Ach) were obtained over the range of 10^−9^ to 10^−5^ mol/L. Relaxant responses were expressed as percentage of maximal contractions of 0.1 µmol/L NE. After repeated rinsings, the tissues were equilibrated for another 30 minutes, cumulative concentration-response curves for NE were obtained over the range of 10^−9^ to 10^−5^ mol/L.

**Figure 5:**
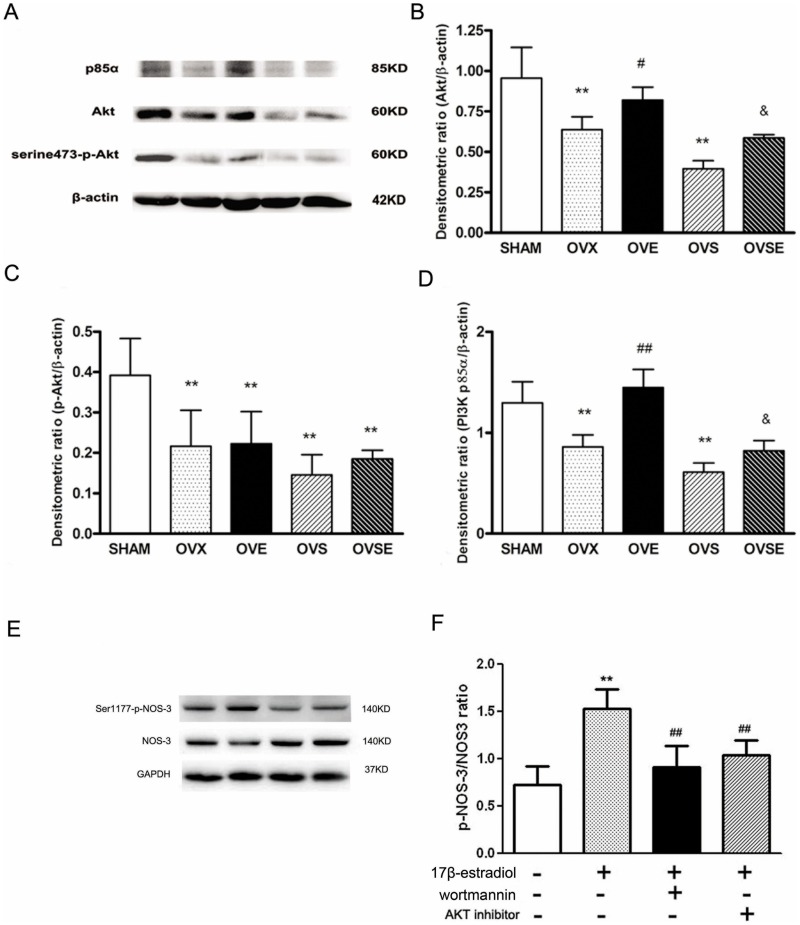
Effects of 17ß-estradiol on PI3K/Akt signal pathway. (A, B) Expression of Akt, (A, C) serine473-p-Akt and (A, D) PI3K p85α in vascular isolated from all groups of animals. (E, F) Expression of serine1177-p-NOS-3 and NOS-3 in EA hy 926 cells pre-incubated with specific PI3K inhibitor (wortmannin, 200 nM) and AKT inhibitor (20 µM) before 17ß-estradiol (100 nM) stimulation. Western blot analysis was performed using each relevant antibody. β-actin and GAPDH was performed as a loading control. Data were shown as mean ± SEM from 6 sets of independent experiments. ^**^ *P*<0.01, vs. SHAM; ^#^ *P*<0.05, ^##^ *P*<0.01, vs. OVX; ^&^ *P*<0.05 vs. OVS in animal experiments.^ **^ *P*<0.01, vs. control; ^##^ *P*<0.01, vs 17ß-estradiol stimulation alone in cellular experiments.

### Cross-sections and Histology

The thoracic aorta sections were gently removed, cleaned, and gradient washed in PBS to 7% saccharose overnight, then were included in OCT compound (Sakura), and 4 µm serial sections were prepared using a cryotome (Leica). Three sections, each separated by 10 sections, were stained with hematoxylin and eosin for each aorta sample. The histological sections were viewed under microscope, and the images were acquired using an attached video camera. For the purpose of comparison, the exact same conditions were used to acquire each image. External and lumen diameter, and intima-media thickness were analyzed as described previously [14].

**Figure 6:**
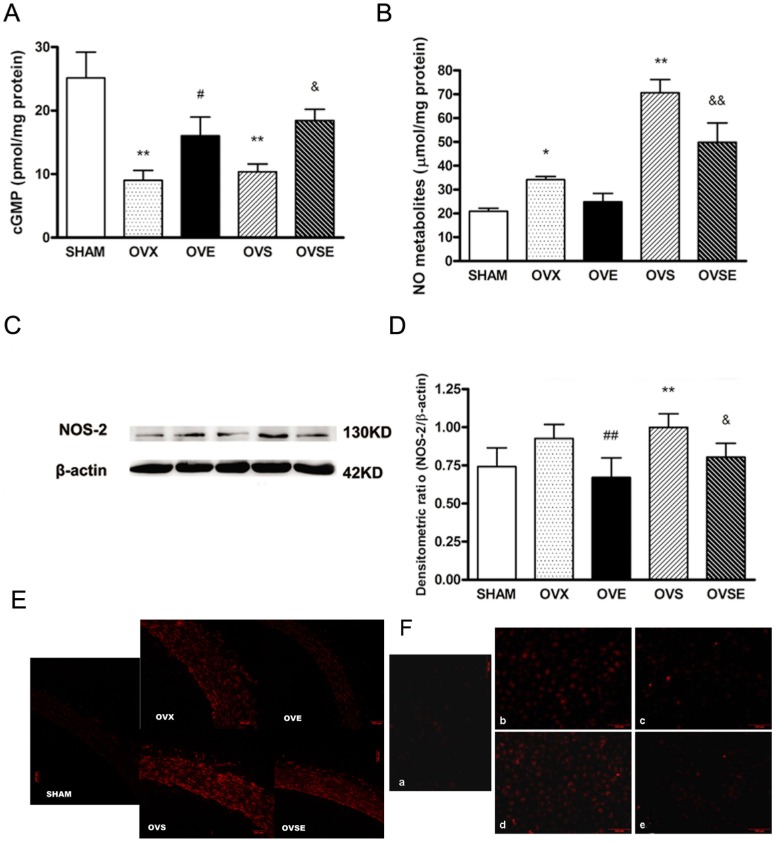
Effects of 17ß-estradiol on NO bioactivity, total NO metabolites, NOS-2 protein expression and ROS production. (A) Effects of 17ß-estradiol on vascular cGMP, (B) NO metabolites and (C, D) NOS-2 (E) ROS production from all groups of animals. (F) Effects of 17ß-estradiol on ROS production in EA hy 926 cells with H_2_O_2_ stimulation. a, control; b, H_2_O_2_(100 µM); c, pre-incubated with 17ß-estradiol (100 nM) 24 hours before H_2_O_2_ (100 µM) stimulation; d, pre-incubated with 17ß-estradiol (100 nM) and specific ER antagonist (ICI 182780, 1 µM) 24 hours before H_2_O_2_ (100 µM) stimulation; e, 17ß-estradiol (100 nM). Data were shown as mean ± SEM from 6 sets of independent experiments. ^*^ *P*<0.05, ^**^ *P*<0.01, vs. SHAM; ^#^ *P*<0.05, ^##^ *P*<0.01, vs. OVX; ^&^ *P*<0.05,^ &&^ *P*<0.01, vs. OVS.

### Extracellular Matrix Marker Measurements

Metalloproteinases and their inhibitors are indices of cardiovascular tissue matrix turnover and arterial stiffness [15], [16]. Thus we determined the arterial contents of MMP-9 and TIMP-1 in the animals to confirm the vascular function and structure variation due to diabetes and estrogen depletion. MMP-9 in both arterial tissue and cell culture medium, TIMP-1 in arterial tissue was measured in duplicate with the use of an ELISA kit (MaiBiotech). The intra-assay coefficient of variation was <5% for the measurements.

### Western Blot Analysis

Equal amounts of protein (50 µg) from the aorta of all groups of animals and EA hy 926 cells were separated by electrophoresis in a 7.5% polyacrylamide-SDS gel and transferred to PVDF membranes (Millipore). After blocking with 5% non-fat milk, membranes were hybridized overnight with primary anti-NOS-3, anti-ERα, anti-phospho serine 118 ERα, anti-PI3K p85α (1∶500, Santa Cruz), anti-phospho serine 1177 NOS-3, anti-phospho serine 473 Akt (1∶500, Cell Signaling) or anti-NOS-2 (1∶500, Bioworld), washed, and incubated with horseradish peroxidase-conjugated secondary antibodies for 2 h at room temperature. After extensive washing, membranes were developed with enhance chemiluminescence reagents (Thermo).

### cGMP and NO Metabolites Determination

Vascular tissue cGMP concentration was quantified by ELISA kits (R&D Systerms) as described previously [17]. The concentration of total NO metabolites (nitrite and nitrate) in vascular tissue was determined with NO assay kits (Byotime), where NO metabolites were detected based on Griess reaction to form a purple azo dye, and then absorbance at 540 nm was evaluated [18].

### Dihydroethidium (DHE) Staining

Reactive oxygen species (ROS) in vessels of rat thoracic aorta was detected by fluorescence microscope using the fluorescent probe DHE. Firstly, aorta was carefully excised and placed in chilled Krebs’ buffer. Connective tissue was removed and segments of upper descending thoracic aorta were frozen in optimal cutting temperature (OCT) compound. Sections (5 µm) were subsequently incubated (30 min, 37°C) in Krebs’ HEPES buffer (mM: NaCl 99; KCl 4.7; MgSO4 1.2; KH2PO4 1.0; CaCl2 1.9; NaHCO3 25; glucose 11.1, NaHEPES 20, pH 7.4) containing DHE (2 µM) in a light-protected chamber.

Following 17β-estradiol stimulation for 24 hours, EA hy 926 cells were incubated with H_2_O_2_ (200 µM) for another 4 hours. Then cells were washed with Krebs’ HEPES buffer, and incubated for 1 hour at 37°C with DHE (5 µM) in Krebs-HEPES buffer. Both slides and cells were examined with a Nikon TE2000 Inverted Microscope (Nikon Ltd., Japan), using excitation and emission wavelengths of 480 and 610 nm respectively.

### Statistical Analysis

Data were expressed as mean±SEM. Data analysis and concentration-response curves were obtained with Prism (GraphPad Software). The comparison between groups was performed by one-way ANOVA followed by Bonferroni’s test as appropriate. Values of *P*<0.05 were considered significant.

## Results

### 17ß-estradiol Regulates Glycometabolism Parameters without Affecting Cholesterol Levels in Diabetic Rats with Ovariectomy

OVS and OVSE animals gained much less body weight as compared to control counterparts, which suggested that 17β-estradiol treatment had little effect on body weight. 17β-estradiol reintroduction in our research produced comparative E2 concentrations with that in SHAM group, and restored uterus weight in OVE and OVSE animals. After 4 weeks of experimental diabetes, OVS and OVSE animals had a significant decrease of plasma insulin, a strong increase of blood glucose and HbA1c. 17β-estradiol treatment seems not be able to modulate these parameters. Moreover, 17β-estradiol reintroduction does not seem to affect the cholesterol profile either (Table 1).

### 17ß-estradiol Improves Thoracic Aorta Reactivity in Diabetic Rats with Ovariectomy

The relaxation and constriction effects of the artery ex vivo were tested using arterial rings isolated from thoracic aorta by obtaining concentration-response curves to Ach and NE. Ach induced full vascular relaxation with comparable efficacy and potency in pre-contracted aortic tissues from SHAM animals and control counterparts (data not shown). Vascular relaxation to Ach was significantly impaired in aortic tissues from OVX rats. Such effects were totally improved in OVE animals after 17β-estradiol replacement. Moreover, aortic tissues from OVS animals presented remarkable decrease on relaxation response to Ach and prolonged peak time to maximal relaxation, and 17ß-estradiol partially improved this impairment (Figure 1A–B).

After relaxation assessment, responses to cumulative concentrations of NE (10^−9^ to 10^−5^ mol/L) were recorded in aortic preparations. Though contraction response curves to NE seemed similar in all groups, peak time to maximum contraction displayed a differential tendency. OVX and OVS animals demonstrated prolonged time to maximal contraction, and estrogen reintroduction could improve such impairment as demonstrated in OVE and OVSE animals (Figure 1C–D).

### 17ß-estradiol Decreases Wall Thickness of Thoracic Aorta in Diabetic Rats with Ovariectomy

Histological analysis of the aorta provided no evidence for visible injury or diameter changes irrespective of ovariectomy and experimental diabetes, suggesting the occurrence of functional rather than morphological impairment of the aorta. However, hematoxylin-eosin staining revealed that the vascular wall of aortas tended to be thicker in OVX and OVS groups than that in SHAM group, and it also tended to be thinner in OVE and OVSE groups, though this might not reach a statistical significance due to limited sets of experiments (Figure 2).

### 17ß-estradiol Reduces Extracellular Matrix Marker MMP-9/TIMP-1 via ER/NOS-3 Pathway

We have demonstrated that the vascular dilation response was exaggerated and the contractile response was attenuated in diabetic rats. Previous findings have clarified that MMP-2 and -9 exacerbate arterial stiffening in diabetes [19]. Evidences also suggest a total increase both in vascular and renal MMP-9 in diabetes and that endothelium may be partially responsible for this [20]. We therefore examined the vascular contents of MMP-9 and TIMP-1 in all groups of animals. OVS animals presented much higher levels of MMP-9, and 17ß-estradiol treatment restored it. Though vascular TIMP-1 did not exhibited significant difference among groups (data not shown), MMP-9/TIMP-1 differed in OVS animals with a significant enhancement. 17β-estradiol reintroduction reduced the MMP-9/TIMP-1 ratio as demonstrated in OVSE animals (Figure 3A–B).

In order to confirm the sequence between ER/NOS-3 and MMP9, we firstly added specific ER antagonist (ICI 182780) before 17ß-estradiol stimulation in EA hy 926 cells. Then phosphorylation and total of NOS-3 were detected. We found that phosphorylation of NOS-3 increased after 17ß-estradiol stimulation, which was consistent with reduction of MMP9 in cell culture medium. And ER antagonist significantly prevented the phosphorylation of NOS-3 after 17ß-estradiol stimulation (Figure 3C–D). Moreover, both ER antagonist and L-NAME attenuated decreasing of MMP9 induced by 17ß-estradiol (Figure 3E).

### 17ß-estradiol Modulates Vascular Protein Expression of NOS-3 and ERα

We detected the vascular protein expression of NOS-3 in all groups of animals. OVX rats exhibited a trend of reduction in aortic NOS-3 protein expression, and 17β-estradiol treatment restored this reduction in OVE animals. As predicted, NOS-3 protein expression in OVS group was markedly reduced, and 17β-estradiol treatment attenuated this reduction as presented in the OVSE group. Phosphorylation of NOS-3 in serine1177 residue also showed reduction in OVX and OVS animals, 17β-estradiol treatment demonstrated a tendency to improve the phosphorylation, but it did not reach significance (Figure 4A–C).

We then examined the role of ERα in the modulation effects of 17ß-estradiol on vascular protein expression of NOS-3 in diabetic rats. Although ERα protein levels tended to become lower in OVX rats, 17β-estradiol treatment resulted in a successful increase in ERα in OVE rats. The remarkable reduction in ERα protein expression in OVS rats was only partially counteracted by 17β-estradiol treatment as presented in OVSE rats. Moreover, phosphorylation of ERα on serine 118 residue showed clear reduction in OVX and OVS animals, and 17β-estradiol may restore such reduction demonstrated in OVE rats, but shown no similar effect in OVSE rats, which might due to the diabetic status (Figure 4A, D–E). But there is no significant difference on ratio of p-NOS-3/NOS-3 between groups (Figure 4F).

### Roles of PI3K/Akt Signaling in the Modulation Effects of 17ß-estradiol on Vascular Expression of NOS-3

Meanwhile, we have also determined the vascular protein expression of NOS-3, thereby we examined the changes in protein expression and serine473 phosphorylation of Akt, the positive regulator of NOS-3, and the regulative p85α subunit of PI3K, a positive upper stream modulator of Akt. The protein expression and phosphorylation of Akt was markedly lower in aorta from OVX and OVS rats. After treatment with 17ß-estradiol, enhanced protein expression of Akt was observed in both OVE and OVSE groups, however, serine473 phosphorylation of Akt could not be fully restored. As predicted, protein expression of PI3K p85α exhibited similar changes with Akt (Figure 5 A–D).

In order to investigate if PI3K/Akt is involved in the protective effect of 17ß-estradiol on vascular function, the EA hy 926 cells were pre-incubated with specific PI3K inhibitor and AKT inhibitor before 17β-estradiol stimulation. We found that both of wortmannin and AKT inhibitor can attenuate the p-NOS-3 augmentation after 17ß-estradiol stimulation, which suggested that PI3K/Akt is the upstream of NOS-3 (Figure 5 E–F).

### 17ß-estradiol Partially Restores NO Bioactivity, Decreases Total NO Metabolites and NOS-2 Protein Expression

NO exerts vascular protective effects by activating guanyl cyclase to increase cGMP (so-called ‘NO bioactivity’) [21]. We examined vascular cGMP concentration in all groups of animals. OVX and OVS rats exhibited significant reduction in cGMP, and 17ß-estradiol only partially increased cGMP content in both OVE and OVSE groups (Figure 6A).

Then we measured the stable NO metabolites, nitrite and nitrate, in vascular tissue of all groups of animals. NO metabolites were remarkably increased in OVX and OVS groups, and 17β-estradiol treatment decreased NO metabolites, as shown in OVE and OVSE groups. The decrease of nitrite contents in OVE and OVSE animals may be associated with a decrease in the production of ROS because of 17β-estradiol treatment [22], [23] (Figure 6B).

We thereby examined the NOS-2 protein expression in vascular tissues of the animals. As predicted, OVS animals presented higher expression of NOS-2, in accordance with the decreased cGMP and increased NO metabolites. And 17β-estradiol treatment indeed lowered NOS-2 expression in both OVE and OVSE rats (Figure 6C–D).

### 17ß-estradiol Decreases ROS Production

Aortic endothelial ROS production was measured by dihydroethidium staining in all groups of animals. Compared with SHAM group, endothelial fluorescence was increased in OVX and OVS rats. Moreover, endothelial ROS production showed an apparent decrease following 17β-estradiol recruitment for 4 weeks in OVE and OVSE rats (Figure 6E).

To verify 17ß-estradiol really affects ROS level in endothelial cells, we used DHE staining in EA hy 926 cells after H_2_O_2_ stimulation. We found that 17ß-estradiol could significantly reduced the H_2_O_2_-induced increase in ROS levels in EA hy 926 cells, which was prevented by specific ER antagonist (Figure 6F). These results demonstrated that 17ß-estradiol could effectively inhibit ROS production in endothelium.

## Discussion

Reports from large randomized trials indicated that postmenopausal hormone replacement therapy (HRT) reduces the risk of diabetes [24], [25]. This is the first demonstration that exogenous estrogen could exert beneficial effects on diabetic vessels via ERα/NOS-3 signaling network in a female diabetic rat model after endogenous estrogen deprivation, and the present findings provides possible mechanisms whereby estrogen exerts such vascular actions. Our research renders ERα and NOS-3 as primary targets for the efficacy of estrogen reintroduction. In addition, this research focuses on the effects of estrogen on the connection of vascular NO pathway and the MMP system, in the context that both of them play key roles in arterial functional and structure remodeling [26].

Endothelium-dependent vasodilatation is generally used as a parameter to assess endothelial function of arteries in different pathophysiologies. Conflicting results indicating normal, impaired, or enhanced endothelium-dependent responses in different models of diabetes have been reported [13], [27]. Endothelial dysfunction has been reported in diabetic people and experimental animals with diabetes [28], [29]. In the present study, estrogen reintroduction improved Ach-mediated dilation, which confirmed the previous findings that estrogen or phytoestrogen could improve the endothelium-dependent vascular dilation in ovariectomized animals. Our results also demonstrated that OVS rats responded much weakly to Ach, and estrogen reintroduction partially improved such response. Because endothelial integrity was not affected by ovariectomy, these results emphasized that endothelial functional impairment exceeded the structural change. Ach-induced relaxation was indeed impaired in OVS rats, moreover, due to vascular MMP activation, this impairment may be again exaggerated. And estrogen reduced MMP-9 via ER/NOS-3 pathway in EA hy 926 cell, which was the possible mechanism of estrogen for improvement of endothelial function.

In the present research, OVX animals presented enhanced NE-mediated vasoconstriction, while estrogen administration in OVE rats diminished this enhancement, which confirmed previous findings that estrogen may decrease vasoconstriction due to improvement of NOS signaling, direct relaxant actions on smooth muscle cells, or decrease of the powerful vasoconstrictor endothelin [30]. Our research also demonstrated that OVS rats expressed contractile response to NE in delayed time, whereas estrogen treatment partially normalized such response. Since MMP/TIMP-1 in diabetic group was greatly enhanced, which may underlie the change of vascular tissue due to degradation of extracelluar matrix, then the enhancement of vasoconstriction responses to NE. These results are in accordance with previous findings that aortic vasoconstriction was augmented in a db/db mouse model [31], [32], while in contrast to the findings that vasoconstriction was impaired *in vivo* in metabolic syndrome rats [33], and in other arteries of STZ-induced diabetes rats [34]. The possible reason of this controversy might be that the impairment of the NOS signaling involved in aortic function in hyperinsulinimic diabetic models is much greater than that in insulin-deficient ones [35].

Estrogen receptors have been identified on both vascular endothelial and smooth muscle cells, providing potential pathways by which estrogen could affect vascular function. Researchers have found that estrogen regulates NOS-3 signaling via ERα, not ERβ in endothelial cells [4], [36], furthermore, ERα expression is modulated by estrogen in human and animals [37], [38], and may be a key determinant of vascular endothelial function in healthy pre- and post-menopausal women. Previous findings have confirmed that the ligand of estrogen and ERα could activate tyrosine kinase c-Src via G protein, then the downstream PI3K/Akt and NOS-3 signaling [39]–[41]. Our data also illustrated that both OVX and OVS animals caused a marked fall in ERα expression and serine118 phosphorylation in vascular tissues, resulting in functional impairment of the ERα/NOS-3 signaling network, consistent with the functional assessment in vascular tissues. Furthermore, estrogen recruitment positively regulated ERα expression and serine118 phosphorylation as presented in OVE and OVSE animals, making it clear that ERα plays key roles on vascular NOS-3 signaling and hence vascular function in this diabetic model. Overall, estrogen depletion induced down-regulation on ERα expression and phosphorylation had profound negative impacts on its signaling network and vascular biology in the background of diabetes status.

It has been shown that estrogen upregulate the expression of the gene for transcription of the NOS-3 in humans [42], thereby increasing the synthesis of nitric oxide. Our results also indicate that estrogen’s beneficial actions in diabetic vessels were enhanced by NOS-3 activation, which was at least partially mediated by one of its major regulative pathway PI3K/Akt. In this research, the increase in NOS-3 and even more so in Akt and PI3K p85α protein expression in response to estrogen replacement inferred the regulation mechanism of estrogen on NOS-3 in this diabetes model. Accordingly, serine1177 phosphorylation of NOS-3 was upregulated by estrogen, indicating a regulation mechanism that both protein expression upregulation and phosphorylation modification involved in the PI3K/Akt/NOS-3 pathway. Both of wortmannin and AKT inhibitor can attenuate the serine1177 phosphorylation of NOS-3 induced by 17ß-estradiol, which suggested that PI3K/Akt/NOS-3 signal pathway plays a key role on estrogen for improvement of NO metabolism and endothelial function.

Impaired NO bioavailability may result in endothelial dysfunction, and it is thought to be a characteristic feature of vascular diseases such as diabetic macroangiopathy [43]. Previous findings suggest that NO production is reduced in diabetes and the decrease of NO may relate to the pathogenesis of diabetic endothelial damage [2], [3]. NO plays a central role in maintaining vascular homeostasis through its effects on endothelial cells, smooth muscle cells, leukocytes, and platelets [44]. Eventually, NO probably represents the most important antiatherogenic defense principle in the vasculature. Our results also demonstrated that the bioactive cGMP was severely diminished, while total NO metabolites were intensively augmented in diabetic rats, and estrogen treatment partially restored both of them. Estrogen treatment has been reported to increase production of the vasodilator nitric oxide but to decrease production of the inducible nitric oxide synthetase [45], [46]. Accordingly, our data proved the protective tendency of estrogen via suppression of NOS-2 on the vasculature of diabetic rats.

Previous studies have displayed a key cause of endothelial dysfunction is ROS generation [47]. In our research, there were serious oxidative stresses after ovariectomy in rats or with H_2_O_2_ stimulation in EA hy 926 cells, which was an important factor of the impairment of the vascular endothelium. After treatment, 17ß-estradiol could significantly reduce the production of ROS to decrease the damage to endothelium.

Our research provides experimental proof of a concept that estrogen administration antagonizes the down-regulation of ERα/NOS-3 signaling in vasculature of female diabetic rats after endogenous hormone depletion. And early reintroduction of estrogen or phytoestrogens to post menopause diabetic patient may produce profound beneficial effects. It remains important to gain a better understanding of the molecular mechanisms that confer cardiovascular protective effects of estrogen in dietetic status.
